# Myocardial T1 and T2 mappings with automatic motion correction at 3 Tesla MR: comparison of T1 and T2 measurements by breathhold, free-breathing and cardiac cycle

**DOI:** 10.1186/1532-429X-15-S1-P104

**Published:** 2013-01-30

**Authors:** Yon Mi Sung, Hwanseok Yong

**Affiliations:** 1 Department of Radiology, Gachon University Gil Hospital, Incheon, Republic of Korea; 2 Department of Radiology, Korea University Guro Hospital, Seoul, Republic of Korea

## Background

T1 and T2 mappings are novel quantitative methods to assess microscopic changes in the myocardium. The aim of this study was to assess differences in myocardial T1 and T2 measurements with automatic motion correction according to breathhold, free-breathing and cardiac cycle.

## Methods

Myocardial T1 and T2 mapping at 3 Tesla MR was obtained from 24 healthy volunteers (16 males and 8 females, mean age, 31±4 years). Shortened modified look-locker inversion recovery was used for T1 mapping and T2-prepared single-shot steady-state free precession was utilized for T2 mapping. Automatic motion correction was done by an elastic registration algorithm based on estimating motion-free synthetic images. For each patient, T1 and T2 mapping were performed consecutively at three short-axis levels in mid-diastole during breathhold (BH), in mid-diastole during free-breathing (FB), and in end-systole during breathhold (SYS-BH). T1 and T2 values were measured in six segments per each level. Mean and standard deviation of slice-averaged myocardial T1 and T2 values were compared between BH, FB and SYS-BH.

## Results

Mean slice-averaged myocardial T1 and T2 values displayed good agreement between BH, FB and SYS-BH (T1 values, 1127 vs. 1146 vs. 1123; T2 values, 41 vs. 42 vs. 41). Standard deviation of T1 measurements was significantly different between BH and SYS-BH (p=0.006) and between FB and SYS-BH (p<0.000) but no significant difference was found between BH and FB (p=0.753). Standard deviation of T2 measurements was not statistically different between BH, FB and SYS-BH (p=0.068). Standard deviation of T1 and T2 measurements was lowest in SYS-BH followed by BH and FB.

## Conclusions

Automatic motion correction was effective for both myocardial T1 and T2 quantification. Mean slice-averaged T1 and T2 relaxation times were not different according to breathhold, free-breathing and cardiac cycle. Standard deviation of T1 measurements was significantly reduced in SYS-BH. Variability of myocardial T1 and T2 quantification may be reduced by obtaining in end-systole during breathhold, particularly for T1 mapping.

## Funding

No financial support.

**Table 1:** Comparison of T1 and T2 measurements

	Mean			p value
		
	BH	FB	SYS-BH	
T1 values	1127	1146	1123	0.121

T2 values	41	42	41	0.331

	Standard deviation (SD)			p value
		
	BH	FB	SYS-BH	

T1 values	29.5	33.1	19.6	0.000

T2 values	2.3	2.7	1.9	0.068

BH, in mid-diastole during breathhold; FB, in mid-diastole during free-breathing; SYS-BH, in end-systole during breathhold

